# Social Cognition in Patients With Hypothalamic-Pituitary Tumors

**DOI:** 10.3389/fonc.2020.01014

**Published:** 2020-07-02

**Authors:** Jale Özyurt, Aylin Mehren, Svenja Boekhoff, Hermann L. Müller, Christiane M. Thiel

**Affiliations:** ^1^Biological Psychology Laboratory, Department of Psychology, School of Medicine and Health Sciences, Carl von Ossietzky Universität, Oldenburg, Germany; ^2^Department of Pediatrics and Pediatric Hematology/Oncology, University Children's Hospital, Klinikum Oldenburg AöR, Oldenburg, Germany; ^3^Research Center Neurosensory Science, Carl von Ossietzky Universität, Oldenburg, Germany; ^4^Cluster of Excellence “Hearing4all”, Carl von Ossietzky Universität, Oldenburg, Germany

**Keywords:** craniopharyngioma, brain tumor, hypothalamus, limbic system, social cognition, theory of mind, emotion recognition

## Abstract

**Objectives:** The current study aimed to investigate whether childhood-onset craniopharyngioma patients are impaired in social-cognitive skills, and whether individual differences in task performance are modulated by the neurohormone oxytocin.

**Study design:** We tested 31 adamantinomatous craniopharyngioma patients with and without hypothalamic lesions and 35 age- and gender-matched healthy controls. To test for between-group differences in social-cognitive skills, we experimentally assessed participants' abilities to interpret social signs or dispositions and to understand others' thoughts, feelings, and intentions. Associations between fasting oxytocin saliva concentrations and task performance were analyzed across the whole group of participants.

**Results:** Compared to controls, patients with hypothalamic lesions were significantly less able to identify the correct emotional content of vocal expressions and to understand others' mental states. Judgements of trustworthiness were not different between patients and controls. There were no correlations between the primary measures of task performance and fasting oxytocin saliva concentrations.

**Conclusions:** This is the first study to show that craniopharyngioma patients with hypothalamic lesions are impaired in some aspects of social cognition, which are of high relevance for everyday social interactions. These deficits suggest a disruption of the normal functionality of hypothalamic-fronto-limbic networks and/or additional areas of the social brain, which are at particular risk by hypothalamic location of the tumor and its treatment.

## Introduction

The term social cognition refers to psychological processes, which underlie social behavior, enabling individuals to successfully interact with conspecifics and to navigate in social environments. It includes more basic operations, such as recognizing dispositions and emotions of others (e.g., from vocal or facial expressions or biological motion), and high-level representations, enabling to read others' mental states, such as feelings, beliefs, intentions, and desires (1). Social cognition is dependent on interacting large-scale networks in the brain, whose integrity can be compromised by psychiatric or neurological disorders (2, 3). Other than neurocognitive deficits, which are increasingly considered in brain tumor patients and tested with standardized test batteries, social-cognitive deficits have been widely neglected and have only recently gained attention (4–7).

Craniopharyngiomas are rare intracranial tumors with an incidence of 0.9-2 cases per million persons per year. 30–50% of the cases are diagnosed during childhood and adolescence (8). Due to their location and growth patterns, the histologically benign tumors (WHO Grade 1) represent a significant risk for the integrity of the hypothalamus and associated fronto-limbic networks, which are key components of the social brain (2, 9, 10). At diagnosis, craniopharyngiomas can be located anywhere along an axis extending from the sella turcica, through the pituitary stalk, the optic chiasm, and the hypothalamus (11). Frequent symptoms, which can seriously limit patients' physical and psychosocial functioning, include visual field defects, loss of homeostatic control, hypothalamic obesity, and endocrine and neurobehavioral deficits. The most consistent findings in the cognitive domain are impairments in learning and episodic memory (12). In the social-emotional domain, frequently reported abnormalities include depression, anxiety, mood swings, emotional outbursts, irritability, aggressiveness, and social withdrawal (13, 14). Hypothalamic involvement of the tumor and/or treatment-related hypothalamic lesions are present in the majority of patients with childhood-onset craniopharyngioma (CP) and have major impact on prognosis (15). Social-cognitive impairments have not been considered in CP yet. Clinical observations of patients registered in the German Childhood Craniopharyngioma Registry, however, indicated that some patients with postoperative hypothalamic lesions (HL) suffer from deficits in social-cognitive functioning. Moreover, patients' close others complained about reduced empathy and impairments in social relations, specifically related to social-cognitive skills, such as understanding others' thoughts and emotional states.

In CP in general, impairments in social-cognitive skills may result from brain changes due to the tumor or its treatment. Even with recent microsurgical methods, frontal, temporal, and limbic areas of the social brain are at risk by commonly employed transcranial surgical approaches via frontal or fronto-temporal routes. Tumor growth may also in some cases compromise the integrity of these brain regions. However, CP with HL are at further risk from adverse changes in hypothalamic-fronto-limbic networks due to the tumor or its treatment. The hypothalamus is known to be essential for basic social-behavioral patterns mediating the satisfaction of drives and urges like mating, sexual behavior, and defensive/attack behavior, which are of vital importance for the survival of the individual and its species (16). It exerts behavioral control via strong, mostly bilateral connections with cortical and subcortical limbic areas, such as the orbitofrontal cortex, cingulum, insula, hippocampus, ventral striatum, amygdala, and thalamic nuclei (17, 18), and this anatomical network is paralleled by hypothalamic functional connectivity in the resting state (19). Damage to the hypothalamus may trigger proximal and distal changes in these connected areas, as brain pathology is likely not confined to a discrete region but is propagated along its axonal pathways to affect connected areas (20). Due to frequently observed tumor growth into the third ventricle, patients with hypothalamic involvement of the tumor also have a much higher risk to develop a hydrocephalus which then may lead to changes in the periventricular brain tissue (21) including brain regions involved in social-cognitive functioning such as the anterior cingulate cortex. In recent imaging studies with CP, findings obtained with diffusion tensor imaging (DTI), positron emission tomography (PET; F-18 fluorodeoxyglucose) and voxel-based morphometry (VBM), indicated changes in the hypothalamus, the medial prefrontal, orbitofrontal, and cingulate cortices, the hippocampus, amygdala, and insula, as well as their connections (9, 10, 22).

In addition, tumor- or treatment-related lesions of hypothalamic-limbic networks in CP may induce changes in the production, release, and central receptor binding of the neurohormone oxytocin. Oxytocin is a hypothalamic neuropeptide, which is mainly synthesized in the supraoptic and paraventricular nuclei of the hypothalamus. For peripheral circulation, it is released into the bloodstream via the posterior pituitary gland, and acts as a hormone to affect physical functions. For central circulation, neurons in the paraventricular nuclei project to various limbic structures such as the amygdala, hippocampus, and nucleus accumbens, which have a high density of oxytocin receptors (23). The oxytocinergic system of the hypothalamus and related limbic networks play an important role in the processing of social stimuli, the modulation of social behavior and learning from social interactions (24, 25).

Compared to patients without HL, CP with HL have a higher risk for additional brain lesions which compromise the social brain and/or the central circulation of oxytocin. Hence, we hypothesized that CP with HL are impaired in social cognitive tasks when compared to CP without HL or to healthy controls. The current study used three experimental tasks to test participants' ability to interpret social signs and to understand others' thoughts, feelings, and intentions. In addition, we hypothesized that social-cognitive task performance is positively correlated with oxytocin saliva concentrations (OSC) across the whole group of participants.

## Materials and Methods

### Subjects

Thirty-one patients and 35 healthy controls participated in the current study. Inclusion criteria for all participants were visual abilities not interfering with task performance and a minimum age of 7 years for the Trustworthiness of Faces task, and 12 years for the two remaining tasks. Additional inclusion criteria for healthy controls were absence of psychiatric or neurological disorders, no substance abuse or use of psychoactive drugs, and a maximum age of 40 years to match the age range of the CP. For CP, a reference-confirmed diagnosis of childhood-onset craniopharyngioma was an additional inclusion criterion. Note that one patient prematurely aborted the experiment and another patient was excluded from final data analyses due to a histological diagnosis of xanthogranuloma, which we learned later. One healthy control was excluded due to stimulant medication use not known before data acquisition, leaving 29 CP and 34 healthy controls for analyses. The study was conducted during an annual meeting of the German Craniopharyngioma Support Group, where all patients and healthy controls willing to participate and fulfilling inclusion criteria, were offered to join the experiments. It was part of a larger study that also investigated CP and healthy controls for (i) OSC before and after a standardized breakfast (26), and (ii) the association between OSC, body mass index (BMI), and eating behavior (27). As only 13 age- and gender-matched healthy controls could be recruited during the Support Group meeting, additional controls were recruited by local advertisements to participate in the same procedures at the University of Oldenburg.

In patients, the histological diagnosis of craniopharyngioma was confirmed by reference assessment in all cases. Tumor size was calculated using the formula “1/2 (A x B),” where A, B and C are the maximum dimensions in the standard planes: axial (cranio-caudal, A) and coronal (transverse, B). Tumor- or treatment-related HL were assessed based on a previously published grading system (28) by using the most recent postoperative MR images, medical records detailing the preoperative status, and details on surgical procedures. Patients were assigned to one of three grades depending on the extent of hypothalamic involvement (grade 0, no HL; grade 1, lesion of the anterior hypothalamus, not involving the mammillary bodies and the hypothalamic area beyond the mammillary bodies; grade 2, HL of the anterior and posterior hypothalamic areas, involving the mammillary bodies and the area beyond the mammillary bodies). These assessments were performed by one of the authors (HLM) and/or by a neuroradiologist. To assess the degree of obesity, we first determined the body mass index, for which weight is adjusted for height. In the next step we calculated a reference standard which enables to account for age and sex: the BMI standard deviation scores (BMI SDS) (29). The study was approved by the local standing committee on ethical practice and written parental and/or patient consent was obtained in all cases.

### Experimental Paradigms on Social Cognition

**Figure 1:**
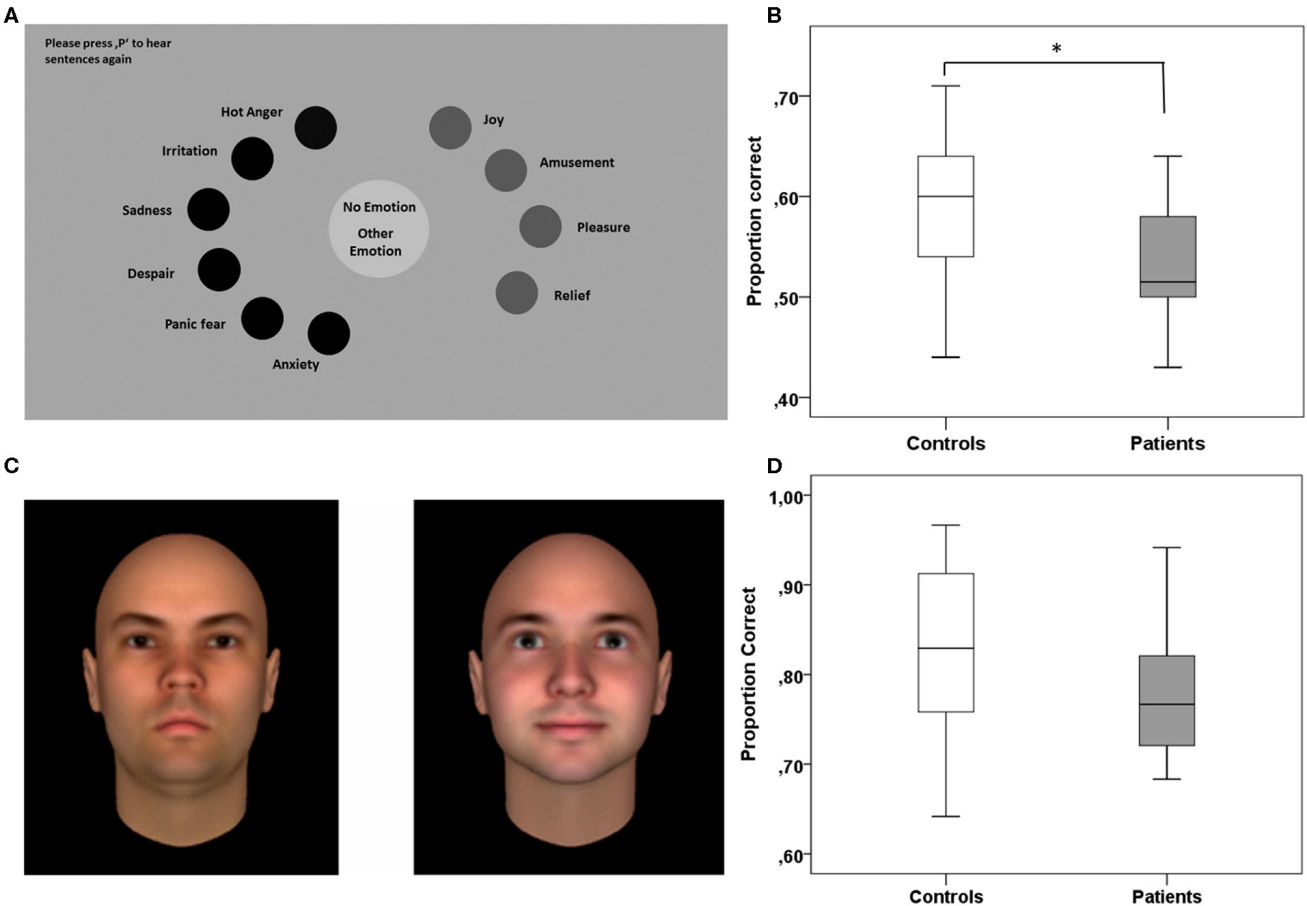
Experimental paradigms testing for specific aspects of social cognition. **(A)** The Identification of Emotional Expressions in Voices was tested with verbal stimuli from the Geneva Multimodal Emotion Portrayals (GEMEP) corpus (30). **(B)** Patients with HL compared to healthy controls were less able to correctly assign emotional expressions in voices to the respective emotion or to the correct emotion category (family accuracy score). **(C)** Trustworthiness of Faces was tested with a validated dataset of computer-generated faces (reproduced with the kind permission of A. Todorov) (32). In each trial of the task, participants had to assign which of two faces presented they found more trustworthy. **(D)** Patients with HL did not differ from controls in assessing the trustworthiness of faces across all trials of the task. *p < 0.05.

The Identification of Emotional Expressions in Voices was tested with verbal stimuli from the Geneva Multimodal Emotion Portrayals (GEMEP) corpus (30). The subset selected comprised 10 core emotional expressions (joy, pleasure, amusement, relief, hot anger [rage], cold anger [irritation], panic fear, despair, anxiety, and sadness). All emotional expressions were embedded in two pseudo sentences, which sound similar to a real but unknown language. The sentences consisted of meaningless words spoken by 10 professional theater actors. The experiment was run on 15.4-inch laptops (resolution 1,440 × 990), and the paradigm was programmed in Matlab using the Psychophysics Toolbox (31). Each trial started with the presentation of a simplified version of the Geneva Emotion Wheel, which was adapted for the subset of stimuli chosen for our experiment (Figure 1A). During the presentation of the emotion wheel, sentences were presented via headphones, and participants were instructed to assign, by a mouse-click, the emotional expression perceived to the corresponding expression depicted on the emotion wheel. Participants had no time pressure and could replay the sentences as often as needed by pressing the letter P on the keypad to then proceed to the next trial by pressing the button “Enter.” A total of 100 trials were presented during the experiment, 10 trials for each of the emotions used. The whole experiment lasted 20 to 30 min, depending on the individual speed of operation. To assess task performance, we used the family accuracy score as the primary measure (30). This score indicates the rate of trials in which patients correctly identified the emotion category, i.e., including cases where confusions occurred between emotions of the same emotion category. For this analysis, we used the emotion categories suggested by Banziger et al. (30): anger (irritation, rage), anxiety (anxiety, panic fear), sadness (sadness, despair), relief (relief), and joy (pleasure, joy, amusement). The rate of correct responses to single emotions are in addition provided in **Table 2**.

#### Trustworthiness of Faces

The ability to assess the trustworthiness of faces was tested with a validated dataset of computer-generated faces (Social Perception Lab, Princeton University, New Jersey, USA; http://tlab.princeton.edu/databases/) (32). The dataset comprises 25 identities of adult Caucasian males, each with 7 levels of trustworthiness (neutral and one to three standard deviations above and below a neutral face). The subset chosen for the current experiment included all levels of trustworthiness except the neutral faces. Thus, we used combinations of faces differing in two to six standard deviations, and each of these combinations was shown 24 times. Patients performed in 120 trials and each trial started with two faces presented side by side at the center of the screen (see Figure 1C). In every case, one of the two faces had a low level and the other a high level of trustworthiness. Faces with low or high trustworthiness were presented on the left or right side of the screen in a randomized order. Participants were instructed to indicate by button press which of the two faces they perceived to be more trustworthy. The face pairs remained on the screen until a decision was made without time pressure. The button press was then followed by the next trial. The task lasted 10–15 min, depending on the individual speed of operation. We used the rate of correct trustworthiness estimations (correct evaluation rate) as the primary measure of the task (**Table 3**).

#### Movie for the Assessment of Social Cognition (MASC)

To assess cognitive empathy, we used the MASC, a video-based test for the assessment of social-cognitive competencies, which can be used with children ≥ 12 years (33). The test requires subjects to watch a movie about four young characters spending an evening and getting together for a dinner. The movie is about everyday social interactions and is paused 46 times after predefined sequences for questions about the actors' thoughts, feelings, and intentions. Participants are required to perform in a multiple-choice format to choose the correct one out of four possible answers. The correct answer is presented with three distractors: answers indicating overly complex mental state reasoning (exceeding theory of mind [ToM] or overmentalizing), overly simplistic mental state inferences (less ToM), and complete lack of mental state inferences (no ToM). Six control questions are also included to allow for the assessment of non-social inferencing capacity. The total duration of the movie sequences adds up to 15 min. The total duration of the testing including stops and continuing in a self-paced manner comprises 30–45 min. In accordance with the observation that CP with HL exhibit overly simplistic mental state inferences (less ToM) or even complete lack of mental state inferences (no ToM), the primary measure used was the sum of cases where participants chose answers indicating less or no ToM (reduced Tom). For a more complete picture, we also provide the rate of all incorrect responses, overmentalizing, and incorrectly answered control questions in **Table 4**.

### Patient- and Treatment-Related Variables

To assess if symptoms of depression or anxiety, which are generally more prevalent in CP compared to controls (14), modulate task performance, we used German versions of two standardized self-report inventories. The Beck Depression Inventory (BDI-II) (34) is a 21-item self-report measure of depressive symptoms experienced during the past 2 weeks. Higher scores indicate higher levels of depressive symptoms and scores are categorized as follows: minimal (9–19), mild (14–19), moderate (20–28), and severe level of depressive symptoms (29–63). The State Trait Anxiety Inventory (STAI) (35) is a 40-item measure to assess trait and state anxiety by using two subscales with each 20 items. As the STAI has no accepted categorization of the severity of anxiety, we used +1.5 standard deviations as a cut-off value to characterize more severe symptoms. In our sample, the minimum raw score, which exceeded + 1.5 standard deviations, ranged from 49 to 53, depending on age and sex. In both questionnaires, items are rated on a 4-point Likert scale; from 0 = not at all to 3 = severely.

For the primary measures of all experimental tasks, we assessed whether radiation therapy and age at diagnosis were associated with task performance. For the two tasks lasting longer than 15 min (Identification of Emotional Expression in Voices and MASC), we tested in addition whether patients' performance decreased over time to a stronger extent than that of controls.

### Oxytocin Analysis in Saliva

OSC were measured under fasting conditions as well as directly after a standardized breakfast. Saliva was collected from each participant using a standardized method for saliva sampling. A white cylindrical swab was chewed gently for 15 min by each participant and then returned with the absorbed saliva into a salivette. All salivettes were placed immediately on ice and kept cool until centrifugation. After centrifugation, patient saliva samples were stored frozen until analysis. A protease inhibitor such as aprotinin was not used after centrifugation. OSC were measured by immunoassay (36). Briefly, a competition between biotinylated oxytocin used as tracer and endogenous oxytocin provides a displacement curve after incubation with streptavidin-horseradish peroxidase and revelation with TMB 3,3',5,5'-Tetramethylbenzidine. Filtration on Amicon Ultracel-2, membrane of 3 kDa (cutoff 3000) - both from Merck Millipore - was performed in order to reduce interferences. Filtration recovery was measured as 68.4 ± 14.4% (coefficient of variance: 21%) of 10 pg added to hormone-free plasma. Combined intra-assays standard deviations (SD) for the enzyme immunoassay (EIA) (*n* = 10 in duplicates and triplicates) were 1.05% at 7.2 pg/ml, 1.94% at 20 pg/ml, and 1.58% at 200 pg/ml. Detection limit (± 2 SD from zero) was typically 0.5 pg/ml and always under 1 pg/ml. We correlated fasting OSC scores with each of the primary task performance measures to analyze associations between OSC and task performance. We also correlated fasting OSC scores with state anxiety and depression scores, respectively, to reproduce the well-known negative associations between these variables [e.g., Ladegaard et al., (37)]. All these correlations were performed across the whole group of participants.

### Statistical Procedures

Our initial aim was to compare task performance (i) between CP with HL and healthy controls, and (ii) between CP with and without HL. However, only few patients without HL participated in the experiments and due to parallel events in the schedule of the Support Group Meeting, some of the participants only performed in one or two of the three tasks. This resulted in a low number of patients without HL in the individual tasks (ranging from *n* = 4–5). For this reason, our analyses focused on the comparison of task performance between patients with HL and healthy controls. In addition, as most studies focusing on neurobehavioral performance in CP have used mixed groups with and without HL, we also show, for comparability, results for the comparison of all CP vs. healthy controls. Statistical analyses were performed with SPSS 23® for Windows (Armonk, NY: IBM Corp.). Due to small sample sizes and non-normal distribution of the data, we chose to apply non-parametric methods. For between-group comparisons of categorical variables, we used Fisher's exact test. For all other between-group comparisons (except distribution analyses), we used the Mann–Whitney *U*-test. To test for bivariate correlations, we used the Spearman correlation coefficient. To examine whether patients' performance in a task decreased over time differently when compared to healthy controls, we applied the following procedure: For each group, errors in each trial were summed up across participants and error rates for each trial were then computed as a proportion of all errors within a group. In a second step, the cumulative error rate distributions were compared between groups by using the Kolmogorov-Smirnov test for two independent samples. Note that with the exception of the primary measures predefined for each of the three experimental tasks, all analyses were explorative. The significance level for all tests was set to *p* < 0.05 (two-sided).

## Results

Subject characteristics for the total groups of patients and healthy controls as well as for the subgroups of patients with HL (*n* = 22) and without HL (*n* = 7) are depicted in Table 1. Of all patients with HL, 22.7% had a lesion confined to the anterior part of the hypothalamus (grade 1, *n* = 5) and 77.3% had a lesion of the anterior plus the posterior hypothalamus (grade 2, *n* = 17). The total group of patients and patients with HL were not significantly different from controls with respect to age, gender, and fasting OSC scores. However, they differed significantly from controls with respect to BMI SDS and had numerically higher depression and anxiety scores. Categorization of the BDI-II and STAI results revealed that none of the patients had scores indicating moderate or severe symptoms of depression and only four CP with HL had STAI trait scores, which exceeded +1.5 standard deviations when compared to STAI norms. As the STAI and BDI-II are not appropriate for ages <15 and <13 years, respectively, scores were not obtained for the youngest participants. For OSC analysis, saliva was collected from all but three participants (one patient) who decided not to participate in this procedure. An additional five salivates (four patients) could not be analyzed due to insufficient amounts of saliva. Twelve of the patients had received local external irradiation, 40.9% of the patients with HL (*n* = 9) and 42.9% of the patients without HL (*n* = 3). Irradiation was performed with doses ranging from 45 to 54 Gy. Two patients received a single dose stereotactically guided convergent beam radiotherapy (LINAC, 12 Gy and Gamma-Knife, 10 Gy, respectively). The median time from irradiation in the patient sample was 7.8 yrs. (IQR: 10.8; range: 2 months to 20.8 yrs.).

**Table 1:** Demographic data for the total group of patients, the two subgroups of patients with and without postoperative hypothalamic lesions (HL), and the healthy control group.

	**All patients^a^**	**Patients without postop HL**	**Patients with postop HL**	**All controls**	**All patients vs. controls (*p*-value)**	**Patients without HL vs. with HL (*p*-value)**	**Patients with HL vs. controls (*p*-value)**
N	29	7	22	34			
% Female gender	58.6	57.1	59	55.9	0.803	1	0.8
Age at study	20 (11) range: 7–38	20 (11) range: 7–32	19.5 (10) range: 12–38	23.5 (13) range:8–40	0.26	0.74	0.36
Age at diagnosis	10.9 (6.6)	9.5 (7)	11 (7)	–	–	0.098	–
BMI SDS	4.3 (6.6)	1.6 (1.1)	5.5 (5)	0.42 (1.5)	<0.001^***^	0.008^**^	<0.001^***^
% Radiation therapy	41,4	42.9	40.9	–	–	1	–
n Baseline OSC	24	6	18	31			
Baseline OSC	3.3 (3.6)	2.7	3.61 (3.3)	4.42 (7.6)	0.329	–	0.474
n BDI-II	21	5	16	31			
BDI-II raw scores	6 (11)	6	6 (10)	4 (5)	0.091	–	0.051
% With moderate/severe depressive symptoms in BDI	0	0	0	2.9/0	–	–	–
n STAI	22	5	17	30			
STAI state	34.5 (16)	34	36 (17)	31.5 (6)	0.04	–	0.092
STAI trait	40 (16)	36	40 (16)	35.5 (8)	0.121	–	0.061
% exceeding +1.5 SD in STAI trait	13.8	0	18.2	2.9	–	–	–

*Medians and interquartile ranges (IQR, in brackets) are depicted, unless otherwise specified. For n <7 (see patients without hypothalamic lesions), IQR and statistical test results were not computed. With the exception of gender (χ2- test), all analyses were performed with the Mann-Whitney U-test*.

* p < 0.05;

** p < 0.01;

*** *p < 0.001*.

*BMI SDS, body mass index standard deviation scores; OSC, oxytocin saliva concentration. BDI, Beck Depression Inventory; STAI, State-Trait-Anxiety Inventory*.

a *including patients with and without postoperative HL*.

Information on the composition of the tumor was available for 72.4% patients (*n* = 21) and classified as cystic in 4 and both cystic and solid in 17 patients. Tumor size in 2D was specified for 62.1 % of the patients (*n* = 18), with a median size of 8.1 cm^2^ (IQR = 13.38) ranging from 0.25–9.61 cm^2^ for patients without HL (*n* = 4) and 1.56 cm−47.12 cm^2^ for patients with HL (*n* = 14).

Information on surgical approaches was available for 75.9% of the patients (transcranial surgery, *n* = 20, transsphenoidal surgery, *n* = 2). Details of the transcranial approach were specified for 18 patients and included right pterional (*n* = 8), subfrontal (bifrontal or frontolateral, *n* = 9), and transcallosal (*n* = 1) surgeries. Data on completeness of the tumor resection was available for 86.2% of the cases, with 11 complete resections and 14 incomplete resections. Data on absence or presence of a hydrocephalus was available for 69% of the patients (*n* = 20). Seven of these patients presented with a hydrocephalus and all of these patients had a postoperative HL.

Imaging findings from the patient records were available for 82.8% of the patients (*n* = 24). For the frontal lobes, they revealed a high percentage of cases with often small but lasting abnormalities (54.2%, *n* = 13, >6 months), frequently visible in the surgical canal or its vicinity (described, for example, as substance defect, gliosis, hygroma). Three of the lasting defects were associated with the tumor itself growing into frontal lobe areas. Apart from these lasting defects, frontal abnormalities in three patients were only reported to be visible in the early postoperative phase. Postoperative changes in the striatum (*n* = 2), the striatum and thalamus (*n* = 1), the temporopolar region (*n* = 2), the temporal operculum (*n* = 1), and the temporooccipital cortex (*n* = 1) were only observed in single cases. Preoperative compression of the brainstem by the tumor was seen in two patients and abnormalities in the centrum semiovale was reported for one patient.

### Social Cognitive Performance

Subject characteristics and performance data for each of the tasks are displayed in Tables 2–4.

**Table 2:** Experimental task results and demographic and clinical variables for patients with postoperative hypothalamic lesions (HL) and controls participating in the Identification of Emotional Expressions.

**Identification of Emotional Expressions**					
	**All patients^a^**	**Patients with postop HL**	**All controls**	**All patients vs. all controls (*****p*****-value)**	**Patients with HL vs. all controls (*****p*****-value)**
**Task Performance**					
n	16	12	29		
Single emotions	0.38 (0.12)	0.35 (0.12)	0.43 (0.11)	0.045^*^	0.039^*^
**Family accuracy score**	0.525 (0.13)	0.515 (0.11)	0.6 (0.11)	0.061	0.039^*^
Family accuracy score (negative items)	0.563 (0.16)	0.517 (0.13)	0.567 (0.13)	0.075	0.046^*^
Family accuracy score (positive items)	0.563 (0.16)	0.563 (0.14)	0.625 (0.14)	0.224	0.216
**Demographic and Clinical Variables**					
n	16	12	29		
% Female gender	56.3	75	62.1	0.758	0.507
Age at study (years)	22.5 (14) range: 13–38	22.5 (16) range: 13–38	24 (12) range: 12–40	0.766	0.989
Age at diagnosis (years)	10.9 (5.9)	10.9 (5.6)	–	–	–
BMI SDS	4.5 (7.2)	5.1 (6.4)	0.42 (1.5)	<0.001^***^	<0.001^***^
% Radiation therapy	50	50	–	–	–
n Baseline OSC	15	11	26		
Baseline OSC	3.7 (3)	3.7 (3.1)	4.6 (9.1)	0.336	0.349
n BDI-II	14	11	29		
BDI-II (raw scores)	6 (12)	12 (10)	4 (5)	0.231	0.04^*^
n STAI State	14	11	28		
STAI State (raw scores)	35.5 (11)	37 (12)	31 (6)	0.004^**^	0.007^**^
STAI Trait (raw scores)	40 (17)	43 (10)	35.5 (9)	0.177	0.034^*^

*For the experimental task, the proportion of correctly matched emotions/emotion categories is depicted and the primary measure is highlighted in bold. Medians and interquartile ranges (in brackets) are shown, unless otherwise specified. With the exception of gender where we used the χ2- test, all analyses were performed with the Mann-Whitney U–test*.

* p < 0.05;

** p < 0.01;

*** *p < 0.001*.

*HL, hypothalamic lesion; BMI SDS, body mass index standard deviation scores; OSC, oxytocin saliva concentration; BDI, Beck Depression Inventory; STAI, State-Trait-Anxiety Inventory*.

a *including patients with and without postoperative HL*.

**Table 3:** Experimental task results and demographic and clinical variables for patients with postoperative hypothalamic lesions (HL) and controls participating in the Evaluation of Trustworthiness.

**Evaluation of Trustworthiness**					
	**All patients^a^**	**Patients with postop HL**	**All controls**	**All patients vs. all controls (*****p*****-value)**	**Patients with HL vs. all controls (*****p*****-value)**
**Task Performance**					
n	20	15	32		
**Correct evaluation rate (across all items)**	0.804 (0.15)	0.767 (0.11)	0.829 (0.16)	0.328	**0.135**
Correct evaluation rate (for difficult items)	0.677 (0.17)	0.667 (0.15)	0.74 (0.21)	0.214	0.093
**Demographic and Clinical Variables**					
n	20	15	32		
% Female gender	60	53.3	56.3	1	1
Age at study (years)	20.5 (10) range: 7–38	21 (9) range: 13–38	23.5 (13) range: 8–40	0.770	0.882
Age at diagnosis (years)	10.5 (6.1)	10.9 (6.48)	–	–	–
BMI SDS	3.73 (6.65)	5.51 (6.63)	0.32 (1.53)	<0.001^***^	<0.001^***^
% Radiation therapy	45	46.7	–	–	–
n Baseline OSC	17	13	29		
Baseline OSC	3.73 (2.72)	3.73 (2.70)	4.52 (7.82)	0.352	0.327
n BDI_II	15	12	29		
BDI-II (raw scores)	6 (11)	9 (10)	4 (5)	0.208	0.038^*^
n STAI	16	13	28		
STAI State (raw scores)	36.5 (12)	38 (12)	31 (6)	0.001^***^	0.001^***^
STAI Trait (raw scores)	41.5 (17)	47 (12)	35.5 (9)	0.060	0.008^**^

*For the experimental task, the proportion of correct evaluations is depicted and the primary measure is highlighted in bold. Medians and interquartile ranges (in brackets) are shown, unless otherwise specified. With the exception of gender where we used the χ2- test, all analyses were performed with the Mann-Whitney U-test*.

* p < 0.05;

** p < 0.01;

*** *p < 0.001*.

*HL, hypothalamic lesion; BMI SDS, body mass index standard deviation scores; OSC, oxytocin saliva concentration; BDI, Beck Depression Inventory; STAI, State-Trait-Anxiety Inventory*.

a *including patients with and without postoperative HL*.

**Table 4:** Experimental task results and demographic and clinical variables for patients with postoperative hypothalamic lesions (HL) and controls participating in the MASC.

**Movie for the Assessment of Social Cognition (MASC)**					
	**All patients^a^**	**Patients with postop HL**	**All controls**	**All patients vs. all controls (*****p*****-value)**	**Patients with HL vs. all controls (*****p*****-value)**
**Task Performance**					
n	22	17	31		
n Incorrect ToM	12 (9)	13 (8)	9 (6)	0.138	0.004^**^
**n Undermentalizing (Reduced ToM)**	6.5 (3.8)	7 (3)	3 (4)	0.032^*^	0.001^**^
n Overmentalizing (Excess ToM)	5 (5)	6 (5)	5 (4)	0.736	0.245
n Correct control questions	5 (1)	5 (2)	5 (2)	0.386	0.162
**Demographic and Clinical Variables**					
n	22	17	31		
% Female gender	63.6	64.7	54.8	0.581	0.555
Age at study (years)	20 (12) range: 12–38	18 (13) range: 12–38	24 (12) range: 12–40	0.333	0.261
Age at diagnosis (years)	10.9 (6.3)	10.9 (7.4)	–	–	–
BMI SDS	4.7 (5.1)	5.51 (3.85)	0.42 (1.79)	<0.001^***^	<0.001^***^
% Radiation therapy	36.4	35.3	–	–	–
n Baseline OSC	19	14	29		
Baseline OSC	3.7 (3.8)	3.84 (3.44)	4.42 (8.17)	0.441	0.515
n BDI-II	18	13	30		
BDI-II (raw scores)	6 (11)	6 (11)	4 (5)	0.096	0.062
n STAI State	18	13	29		
STAI State (raw scores)	34 (14)	34 (16)	31 (6)	0.07	0.085
n STAI Trait	18	13	28		
STAI Trait (raw scores)	39 (13)	40 (15)	35 (7)	0.165	0.126

*For the experimental task, number of errors are depicted and the primary measure is highlighted in bold. Medians and interquartile ranges (in brackets) are shown, unless otherwise specified. With the exception of gender where we used the χ2- test, all analyses were performed with the Mann-Whitney U-test*.

* p < 0.05;

** p < 0.01;

*** *p < 0.001*.

*HL, hypothalamic lesion. ToM, Theory of Mind; BMI SDS, body mass index standard deviation scores; OSC, oxytocin saliva concentration. BDI, Beck Depression Inventory; STAI, State-Trait-Anxiety Inventory*.

a *including patients with and without postoperative HL*.

#### Identification of Emotional Expressions in Voices

Compared to healthy controls, patients with HL were less able to correctly assign emotional expressions in voices to single emotions or to emotion categories (family accuracy score as the primary measure of the task; *z* = 2.069 *p* = 0.039; see Figure 1B). For the total group of patients compared to controls, we obtained similar results, albeit significant only for the matching of single emotions (*z* = 2.007 *p* = 0.045). Noteworthy, results obtained for the four patients without HL were similar to those of controls, which implies that results for the total group are driven by the group with HL (Table S1). As depicted in Table 2, explorative analyses revealed that patients with HL performed significantly worse compared to controls, when required to assign negative emotions to vocal expressions (family accuracy score: *z* = 1.996; *p* = 0.046) but not when required to assign positive emotions (family accuracy: score: *z* = 1.253; *p* = 0.216).

#### Assessment of Trustworthiness of Faces

Patients with HL were not different from healthy controls when asked to evaluate the trustworthiness of two faces and to decide which one they find more trustworthy (correct evaluation rate as the primary measure of the task; *z* = 1.496; *p* = 0.135; see Figure 1D and Table 3). An explorative analysis of the more difficult trials, with combinations of faces differing only one to four standard deviations in trustworthiness, revealed worse but non-significant performance differences in patients with HL compared to healthy controls (*z* = 1.681; *p* = 0.093). There were no significant differences between the total group of patients and healthy controls.

#### Movie for the Assessment of Social Cognition

**Figure 2:**
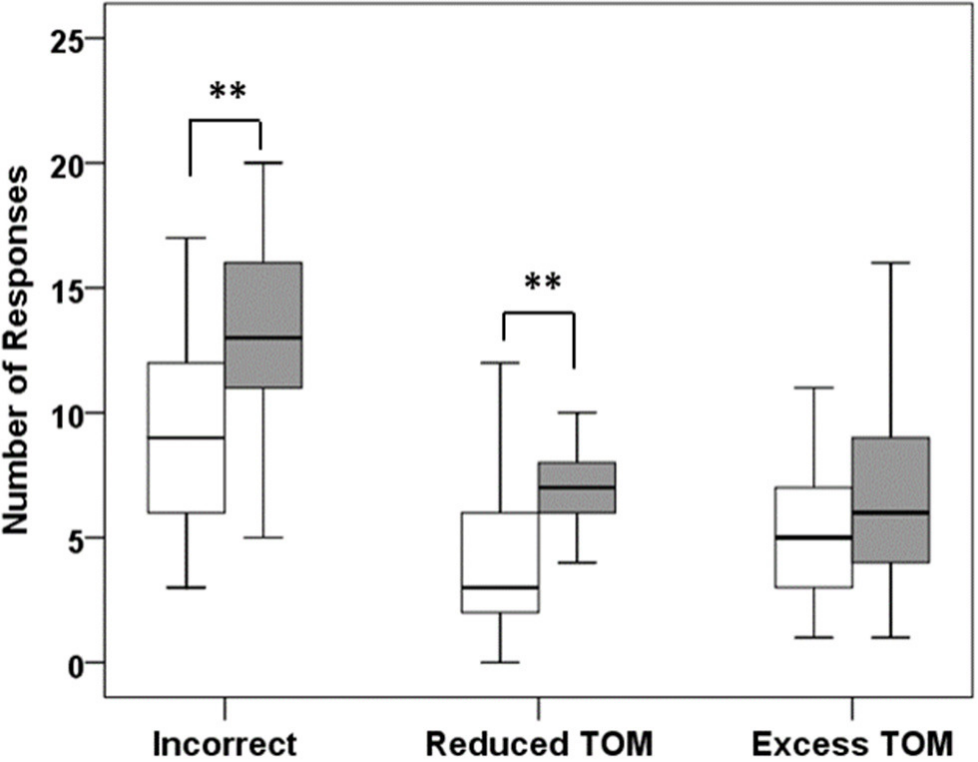
Results of the Movie for the Assessment of Social Cognition (MASC). Patients with HL (dark bars) made significantly more errors than healthy controls (white bars), mainly due to the choice of responses that indicated no or less theory of mind (reduced TOM). ** *p* < 0.01.

Compared to controls, CP with HL significantly more often chose responses indicating undermentalizing, which was the primary measure of the task (reduced ToM; *z* = 3.375; *p* = 0.001; see Figure 2 and Table 4). In addition, they committed more errors indicating incorrect ToM (*z* = 2.875; *p* = 0.004), but did not differ from controls in overmentalizing (excess ToM; *z* = 1.162; *p* = 0.245). Noteworthy, CP with HL and controls were not significantly different in the number of incorrectly answered control questions (*z* = 1.398, *p* = 0.162). However, to account for confounding effects due to deficits in non-social inferencing as far as possible, we additionally performed an analysis after exclusion of participants who answered <4 of 6 control questions correctly (3 healthy controls and 5 patients with HL). This analysis yielded similar results for all measures, e.g., for undermentalizing as the primary measure (reduced ToM: *z* = 2.815; *p* = 0.005). Comparing the total group of CP to healthy controls, patients were observed to have significantly higher scores for undermentalizing (reduced ToM; *z* = 2.15; *p* = 0.032; see Table 4). Note that scores obtained for the five patients without HL were similar to those of healthy controls (Table S1).

**Figure 3:**
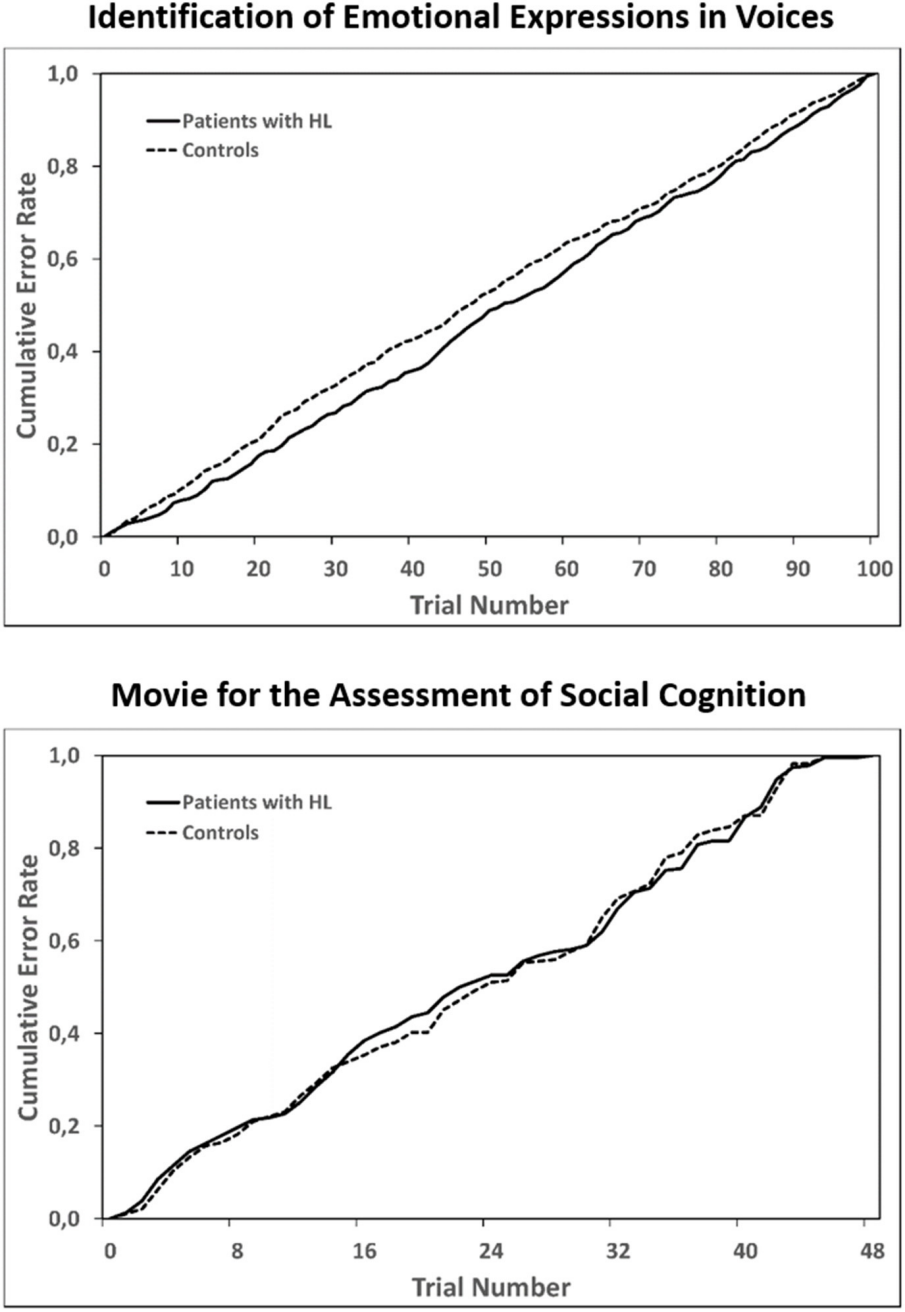
Cumulative error rates across all trials of the two longer tasks: Identification of Emotional Expressions in Voices (upper panel) and the Movie for the Assessment of Social Cognition (lower panel). For each group separately, errors in each trial were summed up across participants and error rates for each trial were then computed as a proportion of all errors within a group. Statistical testing with the Kolmogorov Smirnov test for two independent samples to test for equality of the two distributions in each of the task revealed no significant differences between patients with HL and healthy controls.

For the two tasks which lasted longer than 15 min, statistical analyses revealed comparable cumulative error rate distributions over time when comparing CP with HL to healthy controls (Figure 3; Identification of Emotional Expressions in Voices *p* = 0.906; MASC *p* = 1.0). Furthermore, across the whole patient group, patients who had received radiation therapy were significantly less able to reliably identify the correct emotion category in the Identification of Emotional Expressions in Voices task (*z* = 2.059, *p* = 0.034). Performance in the two other tasks was not significantly different between irradiated and non-irradiated patients.

Additional analyses for gender effects in social cognitive performance, where we included the whole group of participants, revealed no gender differences for the primary measures of the experimental tasks (all *p* > 0.1).

### Individual Differences in Patient- and Treatment-Related Variables and OSC

Across the whole group of participants, the primary measures of our experimental tasks were not significantly correlated with depression and anxiety scores (all *p* > 0.1). Age at study was only correlated with the primary measure of the Identification of Emotional Expressions in Voices task (family accuracy score: *r* = 0.302, *p* = 0.044), but not with those of the Movie for the Assessment of Social Cognition (reduced ToM: *r* = 0.011, *p* = 0.937) and the Evaluation of Trustworthiness task (correct evaluation rate: *r* = 0.115, *p* = 0.419). Within the patient group, none of the primary task measures were correlated with age at diagnosis, age at radiotherapy, or time from radiotherapy (all *p* > 0.1). The degree of hypothalamic involvement was highly correlated with reduced ToM (*r* = 0.743, *p* < 0.001), but not with the other primary task measures.

No significant correlations were found between fasting OSC and the primary measures of the three tasks across the whole group of participants (reduced ToM: *r* = −0.024, *p* = 0.984; family accuracy score: *r* = 0.161, *p* = 0.315; correct evaluation rate *r* = 0.138, *p* = 0.36). However, there was a negative correlation between state anxiety scores and fasting OSC (*r* = −0.327, *p* = 0.027). For depression, there was a significant association of higher symptoms of depression with lower fasting OSC scores (*r* = −0.290, *p* = 0.048).

## Discussion

Intact social-cognitive skills enable individuals to build and maintain stable relations to conspecifics and to take advantage of being part of a social group (1). Accordingly, deficits in social cognition or its components can have far-reaching consequences for patients' every-day lives and vocational opportunities. The current study is the first to investigate social-cognitive performance in patients with hypothalamic-pituitary tumors compared to a healthy control group. The main aim was to investigate whether CP with HL are impaired in specific social-cognitive skills. In addition, we asked whether individual differences in task performance are associated with differences in OSC across the whole group of participants. In accordance with our hypothesis, our main finding was that CP with HL were impaired in specific aspects of social cognition when compared to healthy controls. They were significantly less able to reliably identify the emotional content of vocal expressions, and noteworthy, this result was due to impairment in identifying negative vocal expressions. The correct identification of social signs such as fear and disgust in facial or vocal expressions is particularly important in early child development, to learn about the world from other people and to help avoiding danger (1). By conveying information, which is important for inferences about changing contingencies, facial or vocal expressions also play a significant role in social reinforcement, and hence in adaptive social behavior. Even subtle deteriorations in the ability to identify negative emotional expressions of interlocutors, for example, may have considerable effects upon social relations. Apart from emotion recognition in vocal expressions, we also observed that patients were less able to infer others' thoughts, feelings, and intentions when faced with short film scenes showing social interactions. As expected, mental inferencing in CP was characterized by frequent undermentalizing, whereas overmentalizing was not different to controls. These results also hold when we accounted for deficits in non-social inferencing. Note that the ability to recognize and properly evaluate others' emotional states in speech, facial expressions, or gestures is an essential basis for inferences about others' mental states (38). Importantly, similar cumulative error distributions over time in CP and healthy controls indicated that the higher error rates in patients in the two longer tasks (Identification of Emotional Expressions in Voices and MASC) were not due to an increasingly fatigued state as time on task passed. Facial trustworthiness estimations across all task trials, however, were not impaired in CP compared to controls. However, it should be considered that social trait inferences from faces such as trustworthiness may in part rely on facial expression recognition capabilities, which may confound results obtained with the trustworthiness stimuli used (39).

Social-cognitive deficits in CP with HL are likely due to changes in oxytocin production and release in the hypothalamus and/or structural changes of the hypothalamus, associated fronto-limbic networks, and other brain regions at risk by the tumor and its treatment. Both types of changes of the underlying neurobiological substrate may be induced by the tumor or its therapy.

### Oxytocin Saliva Concentrations

Given the role of oxytocin in social cognition and its production and release in the hypothalamus (25), it is conceivable that the status of the neurohormone and associated behaviors at risk are altered in patients with hypothalamic-pituitary tumors. In a first study on oxytocin in CP, we found that patients were still able to produce and secrete the neurohormone oxytocin into peripheral circulation, even when lesions of the pituitary gland and the hypothalamus were detectable (26). Likewise, in the current study, which used a subsample of this larger study, there was no difference between patients and healthy controls in fasting OSC. Lower fasting OSC in our study were associated with significantly higher state anxiety, as expected from the literature (40, 41). But contrary to our expectations, there were no correlations between OSC and the primary measures of the three social-cognitive tasks. Considering the lack of oxytocin effects in the current study, it should be noted that previous studies investigating oxytocinergic effects on social cognition have almost exclusively used intranasal oxytocin administration, where single doses of oxytocin were found to be sufficient to enhance the accuracy of trustworthiness judgements and the recognition of social signs, like emotional facial expressions or affective speech (42–44). Noteworthy, a recent meta-analysis on correlations between peripheral and central oxytocin concentrations found high positive correlations after intranasal administration but not for baseline conditions, suggesting that peripheral oxytocin measures should not be used as an indicator for central oxytocin levels when investigating social behavior or cognition (43).

### Hypothalamic Networks

Brain abnormalities in CP have only been investigated recently, depicting changes in fronto-limbic brain areas, which are essential for memory, social-emotional, and social-cognitive skills. In a PET-study with CP, a number of tumor- and treatment-related metabolic abnormalities were found in frontal, medial/inferior temporal lobe, limbic areas, thalamus, caudate nucleus and cingulate gyrus (10). Increased intracranial pressure due to hydrocephalus, infarction in caudate or thalamus, cyst catheter tracts, and defects due to surgical pathways were assumed to be the main potential sources of the brain lesions. Similarly, in a study using VBM to characterize tumor- and treatment-related structural brain pathology following a circumscribed lesion to the hypothalamus, we found significantly reduced gray and white matter volumes in patients' fronto-limbic areas connected to the hypothalamus (amygdala, anterior cingulate cortex, ventral striatum, orbitofrontal and medial prefrontal cortex) when compared to healthy controls (9). These brain regions are part of the social brain, supporting social-cognitive skills (2). The ability to accurately interpret others' emotional expressions in face and voice (especially fear) was shown to be particularly impaired with lesions to the amygdala, which is one of the most relevant brain regions for the processing of social-emotional signs (45). Orbitofrontal or ventral anterior cingulate cortex lesions may also impair the identification of affective vocal or facial expressions, and selective impairments in anger recognition were shown in patients with ventral striatal lesions compared to a patient control group with damage to other parts of the basal ganglia (46). Moreover, the anterior rostral cingulate and rostral medial prefrontal cortex, adjacent to the orbitofrontal cortex, were in several studies shown to be involved during social interactions and theory of mind tasks (47). Bilateral lesions to the orbitofrontal cortex in particular were associated with severe changes in social behavior and emotional state (48).

### Patient- and Treatment-Related Factors

Several disease- and treatment-related factors, including surgical approach, obstructive hydrocephalus, peri- and postoperative complications, and radiation effects, may add to tumor-related damage and also result in brain damage outside the region of tumor growth. Concerning postoperative pathology in the patient sample, those with a higher degree of HL in our study presented with a lower ability to infer others' thoughts, feelings and intentions when faced with social interactions. Albeit the degree of hypothalamic involvement is a risk factor for the integrity of hypothalamic-fronto-limbic networks, pathology resulting from frontal and fronto-temporal surgical approaches in CP (irrespective of HL) may significantly add to social-cognitive deficits by harming temporal, medial frontal, inferior frontal and limbic regions. In our study, a high proportion of CP underwent uni- or bilateral frontal or fronto-temporal surgeries, and at least small abnormalities in or in the vicinity of the surgical canal were detectable in many cases. The development of a hydrocephalus, most often associated with HL may be a further risk factor for changes in periventricular regions (21) and associated (social-) cognitive behavior. Apart from surgical approach, irradiation in particular is known to be a risk factor compromising brain tissue and issuing neurocognitive and psychosocial late effects, at least in a subgroup of patients (49). In our study, patients who had received radiation therapy were significantly less able to reliably identify the emotional content in vocal expressions, but performance in the remaining tasks was not different in irradiated compared to non-irradiated patients, which may be due to the small sample sizes of the subgroups.

With respect to patient variables which we accounted for, age at diagnosis, age at radiotherapy, and time from radiotherapy were not correlated with primary task measures, albeit younger age at diagnosis was shown by others to be associated with worse outcomes in neuropsychological performance (49). Further, primary task measures in our study were not significantly correlated with anxiety or depression scores, whereas symptoms of depression and anxiety were shown to be associated with worse social-cognitive performance in previous studies [e.g., Ladegaard et al., (37)].

### Limitations

The current study was limited by relatively small sample sizes and the lack of a brain tumor control group. In addition, there are numerous patient- and treatment-variables which may impact social-cognitive outcomes but could not be controlled for in the current study, including, e.g., intelligence, executive functioning, radiation field, and degree of hydrocephalus. In addition, the generalizability of our results is debatable, due to the selected sample of CP attending a meeting of the support group. However, based on our own knowledge on patients attending this meeting during the past 20 years, and the low rate of patients with more severe symptoms of depression and anxiety in the current study, we speculate that patients who suffer from more severe sequelae are underrepresented in CP attending these meetings. This implies that social-cognitive deficits may be even more severe and frequent in a more representative sample of CP. Finally, it should be noted that the primary measures in our study were experimental in nature. Thus, future studies should relate social-cognitive functioning in patients with brain tumors to daily life measures.

## Conclusions

The ability to correctly interpret others' mental states is crucial for successful social functioning, and impairments significantly challenge families, friends, and the patients' ability to perform in school and working environments [e.g., Pierre-Kahn et al., (13)]. Using experimental tasks, the current study provides initial evidence that CP with HL compared to healthy controls are less able to successfully evaluate social signs and to understand other people's thoughts, intentions, and feelings. Albeit no evidence was found for an association between task performance and oxytocin saliva concentrations, patients' impairments in social-cognitive skills are highly consistent with a perturbation of the normal functionality of limbic, frontal, and temporal areas of the social brain. Such changes may arise from tumor- or treatment-related lesions, which may add to effects of the hypothalamic lesion. Although patients with HL as a group performed significantly worse compared to healthy controls, they show a high individual variability in task performance. We hypothesize that the degree to which patients are impaired in their social skills primarily depends on the type and extent of their lesions to the hypothalamus, as well as on tumor- and treatment-related changes in frontal, temporal and limbic regions. Further investigations with larger samples and additional brain measures are needed to better characterize CP at risk. With regard to clinical practice, there is an urgent need to include tests for social-cognitive functioning in neuropsychological batteries used to test patients with brain tumors. Knowledge on patients' impairments in the social-cognitive domain may serve to partly compensate compromised social functioning by psychoeducation, more informed and supportive environments, and possibly by individually tailored social-cognitive trainings.

## Data Availability Statement

The datasets generated for this study are available on request to the corresponding author.

## Ethics Statement

The studies involving human participants were reviewed and approved by Ethics Committe of the University of Oldenburg, Oldenburg, Germany. Written informed consent to participate in this study was provided by the participants or the participants' legal guardian/next of kin.

## Author Contributions

JÖ and CT conceptualized and designed the study. HM and SB coordinated and supervised patient recruitment. JÖ and AM supervised data collection. HM and SB analyzed and interpreted the clinical data. JÖ and AM carried out final data analyses. JÖ drafted the initial manuscript. CT critically reviewed and revised the initial manuscript. All authors approved the final manuscript as submitted.

## Conflict of Interest

The authors declare that the research was conducted in the absence of any commercial or financial relationships that could be construed as a potential conflict of interest.

## Abbreviations

CP: Childhood Onset Craniopharyngioma Patients
HL: Hypothalamic Lesions
OSC: Oxytocin Saliva Concentration
MASC: Movie for the Assessment of Social Cognition
PET: Positron Emission Tomography
BMI: Body mass index.
