# Supplementary material for: Social Cognition in Patients With Hypothalamic-Pituitary Tumors
Source: Front Oncol. 2020 Jul 2;10:1014. doi: 10.3389/fonc.2020.01014 (PMC7343961; doi:10.3389/fonc.2020.01014)
Supplement: Supplementary file 1 [file Table_1.docx]

Supplementary Material

**Table S1.** Experimental task results for patients without postoperative hypothalamic lesions. As summary statistics are not appropriate for the small sample sizes, single subject scores are depicted. For better comparability to the control group, controls’ medians and interquartile ranges (IQR) are shown for each of the task measures. HL=hypothalamic lesion. ToM=Theory of Mind.

| **Identification of Emotional Expressions** | | | | |
| --- | --- | --- | --- | --- |
| **Patients without HL (n=4)** | **Proportion correctly matched single emotions** | | **Proportion correctly matched emotion categories: Family accuracy score** | |
| **P1** | 0.42 | | 0.59 | |
| **P 3** | 0.42 | | 0.63 | |
| **P 7** | 0.45 | | 0.60 | |
| **P 8** | 0.14 | | 0.24 | |
| **Healthy Controls**  **Median and (IQR)** | 0.43 (0.11) | | 0.6 (0.11) | |
| **Evaluation of Trustworthiness** | | | | |
| **Patients without HL (n=5)** | **Correct evaluation rate**  **(across all items)** | | | |
| **P 1** | 0.90 | | | |
| **P 3** | 0.79 | | | |
| **P 6** | 0.84 | | | |
| **P 7** | 0.93 | | | |
| **P 8** | 0.84 | | | |
| **Healthy Controls**  **Median and (IQR)** | 0.83 (0.16) | | | |
| **Movie for the Assessment of Social Cognition** | | | | |
| **Patients without HL (n=5)** | **n Incorrect ToM** | **n Undermentalizing (Reduced ToM)** | | **n Overmentalizing (Excess ToM)** |
| **P 1** | 3 | 0 | | 3 |
| **P 2** | 4 | 1 | | 3 |
| **P 3** | 6 | 1 | | 5 |
| **P 5** | 4 | 2 | | 2 |
| **P 7** | 10 | 5 | | 5 |
| **Healthy Controls**  **Median and (IQR)** | 9 (6) | 3 (4) | | 5 (4) |
